# Supplementary material for: Using metagenomics and whole-genome sequencing to characterize enteric pathogens across various sources in Africa
Source: Nat Commun. 2025 Nov 28;16:11311. doi: 10.1038/s41467-025-66400-9 (PMC12722760; doi:10.1038/s41467-025-66400-9)
Supplement: Supplementary file 1 — Supplementary Information [file 41467_2025_66400_MOESM1_ESM.pdf]

Supplementary Material for

# Using Metagenomics and Whole-Genome Sequencing to characterize enteric pathogens across various sources in Africa

Cecilie Thystrup*, Tesfaye Gobena, Elsa Maria Salvador, Olanrewaju Emmanuel Fayemi, Happiness Kumburu, Elna M. Buys, Josphat Gichure, Belisário T. Moiane, Dinaol Belina, Ephrasia A. Hugho, Sara Faife, Tosin Segun Ogunbiyi, Gabriel Akanni, Christianah I. Ayolabi, Blandina Mmbaga, Kate M. Thomas, Sara M. Pires, Patrick Murigu Kamau Njage, Tine Hald

*To whom correspondence should be addressed
e-mail: ceth@dtu.dk

**Supplementary Methods:**

**Supplementary Methods 1: Standard Operating Procedures (SOPs) for *Salmonella/Shigella* spp., *Campylobacter* spp., and diarrheaogenic *E. coli* (DEC) methodologies.**

**Standard Operating Procedure for adapted *Salmonella* spp. (and *Shigella* spp.) methodologies**

**<u>*Salmonella* (and *Shigella*) spp. Detection and Isolation Methods: Fluid Milk and Milk Products</u>**

**Introduction:** This Standard Operating Procedure (SOP) was created based upon the FDA BAM and ISO 6579-1:2018 for the detection and isolation of *Salmonella* spp. in fluid milk, milk products and various food stuffs but has been adapted to fit the ENSURE, FOCAL, Pull-Push, and TARTARE projects. The following outlines the procedures for trained personnel working within approved laboratories associated with these project teams. *Shigella* methods are adapted from ISO and FDA-BAM for use in the FOCAL project.

**Safety Precautions**

*Salmonella* and *Shigella* are generally categorized as Biosafety Level 2 pathogens. CDC guidelines for handling Biosafety Level 2 pathogens should be followed whenever live cultures of *Salmonella* or *Shigella* are used. A Class II laminar flow biosafety cabinet is recommended for procedures in which infectious aerosols or splashes may be created. The Safety Data Sheet (SDS) must be obtained from the manufacturer for the media, chemicals, reagents and microorganisms used in the analysis. The personnel who will handle the material should read the SDS prior to startup.

**Equipment and materials**

- Autoclave or sterilization equipment
- Incubators capable of operating at 35 or 37 ± 1 °C and 41.5 ± 1 °C
- Sterile loops 10 µl
- Inoculation needles
- Sterile scalpels
- Sterile cylinders 225 or 500 ml
- Pipettor 20-200 µl
- Pipettor 100-1000 µl
- Sterile pipette tips 1000 µl
- Sterile pipette tips 200 µl
- Sterile serological pipettes 25 ml
- Petri dishes
- Refrigerator (5 ± 3 °C)
- Vortex
- Stomacher (optional)
- Balance (to at least 500 g)
- Filter stomacher bags (whirlpak bags)
- Sterile universal 30 ml tubes
- Sterile universal 9 ml tubes
- 2 ml cryovials or cryobeads
- Biosafety tip autoclave bags

**Standard Operating Procedure for Detection of *Campylobacter* spp.**

***Campylobacter* spp. Detection and Isolation Methods**

**Introduction:** This Standard Operating Procedure (SOP) was created based upon the FDA BAM and Ethiopian standard ES ISO 10272-1:2018 for the detection and Isolation of *Campylobacter* spp. from foods but has been adapted to fit the ENSURE, FOCAL, Pull-Push, and Tartare projects. The following outlines the procedures for trained personnel working within approved laboratories associated with these project teams.

**Safety precautions**

*Campylobacter* are generally categorized as Biosafety Level 2 pathogens. CDC guidelines for handling Biosafety Level 2 pathogens should be followed whenever live cultures of *Campylobacter* are used. A Class II laminar flow biosafety cabinet is recommended for procedures in which infectious aerosols or splashes may be created. The Safety Data Sheet (SDS) must be obtained from the manufacturer for the media, chemicals, reagents and microorganisms used in the analysis. The personnel who will handle the material should read the SDS prior to startup.

**Equipment and materials**

- Autoclave or sterilization equipment
- Incubators capable of operating at 25 ± 1 °C, 30 ± 1 °C and 37 ± 1 °C
- Sterile loops 10 µl
- Inoculation needles
- Sterile scalpels
- Sterile cylinders 225 or 500 ml
- Pipettor 20-200 µl
- Pipettor 100-1000 µl
- Sterile pipette tips 1000 µl
- Sterile pipette tips 200 µl
- Sterile serological pipettes 25 ml
- Serological pipette controller or silicone pipette filler
- Petri dishes
- Refrigerator (5 ± 3 °C)
- Vortex
- Stomacher (optional)
- Scale (to at least 120 g)
- Filter stomacher bags (whirlpak bags)
- Sterile universal 9 ml tubes
- 2 ml cryovials or cryobeads
- Biosafety tip autoclave bags
- Biosafety autoclave bags
- Autoclave tape
- Sample labeling tape (as needed)
- Filters
- Cotton swabs

**FOCAL method of detection for diarrhoeagenic *E. coli* [enrichment adapted from FDA-BAM, PCR adapted from manufacturer's (SSI Diagnositics) instructions]**

**PURPOSE**
*Escherichia coli* are Gram-negative, rod shaped, facultative anaerobic bacteria that are one of the predominant commensal organisms in the human gut. A subset of *E. coli,* with various virulence factors, are known to cause human illness. Pathogenic groups implicated in food- and waterborne illness include Enterotoxigenic *E. coli* (ETEC), Enteropathogenic *E. coli* (EPEC), Enterohaemorragic *E. coli* (EHEC) and Enteroinvasive *E. coli* (EIEC). **ETEC** are characterised by LT (heat-labile) and ST (heat-stable) toxins. **EIEC** do not produce toxins. They are characterised by their highly invasive mechanisms. Both EIEC and *Shigella* will give positive probe and PCR reactions, it is critical that the organisms are identified first as *E. coli*. **EPEC** have an attaching and effacing (A/E) lesion (encoded by the *eaeA* gene) and the lack of *stx*/*vt* toxin genes. **EHEC** are a subset of STEC/VTEC that cause severe human illness. They contain *stx1/vt1* and/or *stx2/vt2* genes along with the A/E lesion *eaeA* gene. See Shiga-toxin producing *E. coli* SOP.

**SCOPE**
This Standard Operating Procedure (SOP) details the procedures to follow for adequately trained staff and students for the isolation of diarrheagenic *E. coli,* from food products/sample types to be tested in sufficiently equipped laboratories for the FOCAL project.

**STANDARD PRECAUTIONS**
Basic laboratory working practises should be followed at all times and all individuals conducting the activities describes should first review and understand the relevant safety manuals for the laboratories where these procedures will take place. Lab coat and appropriate personal protective equipment (PPE) should be worn to perform all of the activities described in this SOP. All individuals undertaking procedures described should follow Good Laboratory Practises (GLP) in a BLS2 laboratory. Laboratory waste must be disposed of with staff and student health and safety in mind following your laboratory SOPs.

**RESPONSIBILITIES**
- The study coordinator is responsible for ensuring all equipment required to follow this SOP is available, safe, and in good working order.
- The field coordinator is responsible for ensuring all relevant meta-data on the samples is recorded when collected, the delivery of samples and sample information to the laboratory, and temporary storage of samples in the project freezer or fridge as appropriate.
- Each study laboratory technician and student is responsible for documenting receipt of samples and ensuring that they are appropriately stored before samples are tested.
- Each study laboratory technician and student is responsible for documenting appropriate information in regards to testing for each sample and recording of test results in the format decided on by the project.

**Standard Operating Procedure for isolation of *Salmonella, Shigella*, *Campylobacter* spp. and diarrheagenic *E. coli* from stool**

**Introduction:** This Standard Operating Procedure (SOP) was created based on *Salmonella*, *Shigella*, *Campylobacter* and diarrheagenic *E. coli* methods for food and environmental samples for the FOCAL project. The following outlines the procedures for trained personnel working within approved laboratories.

**Safety Precautions**

*Salmonella*, *Shigella*, *Campylobacter* and diarrheagenic *E. coli* are generally categorized as Biosafety Level 2 pathogens. CDC guidelines for handling Biosafety Level 2 pathogens should be followed whenever live cultures of *Salmonella*, *Shigella*, *Campylobacter* and diarrheagenic *E. coli* are used. A Class II laminar flow biosafety cabinet is recommended for procedures in which infectious aerosols or splashes may be created. The Safety Data Sheet (SDS) must be obtained from the manufacturer for the media, chemicals, reagents and microorganisms used in the analysis. The personnel who will handle the material should read the SDS prior to startup.

**Equipment and materials**

- Autoclave or sterilization equipment
- Incubators capable of operating at 42 ± 2 °C and 36 ± 2 °C
- Sterile loops 10 μl, 1 μl
- Sterile cotton tipped swabs
- Vortex
- MacConkey agar (MAC) plates
- Hektoen agar (HEK) plates
- Xylose-Deoxycholate agar (XLD) plates
- Modified charcoal-cefoperazone-deoxycholate agar (mCCDA) plates
- Muller-Kauffman Tetrathionate + novobiocin broth (MKTTn) (10ml)
- Rappart Vassiliadis Soya (RVS) (10ml) broth
- Preston broth (10ml)
- CampyGen sachets (microaerophilic gas packs) 2.5 L and 3.5 L
- 2.5 L Anaerobic jars
- 7 L Anaerobic jars
- Sample labeling tape (as needed)
- Marker pens for labelling plates

**Procedure:**

Make MKTTn broth and aliquot into 10 ml aliquots on the day it will be used.
Allow all media to adjust to room temperature before use.
Appropriate positive and negative controls should be included along side samples, to ensure all steps are working.

<u>**DAY 0: Direct plating**</u>

1. Label one of each plate type (MAC, HEK, XLD, mCCDA) for each stool sample.

# Supplementary Methods 2: Standard Operating Procedures (SOPs) for NGS for Illumina machines

## Annex 4: Laboratory Standard Operating Procedure for FOCAL Next Generation Sequencing for the Illumina MiSeq and PacBio Platforms

1. **Purpose**: To describe the standardized laboratory protocol foe the whole genome sequencing of *Salmonella* sp. on the Illumina MiSeq OR PacBio SMRT platforms and the metagenome sequencing (16s rRNA sequencing OR Shotgun metagenome sequencing) of biological samples.

2. **Scope**: To provide the FOCAL group with a single protocol for performing NGS (WGS, Metagenomics), hence ensuring inter-laboratory and/or inter-institution compatibility of laboratory techniques and of generated data.

3. **Definitions**:
   **WGS**: Whole Genome Sequencing
   **NGS**: Next Generation Sequencing
   **PacBio**: Pacific Biosciences
   **SMRT**: Single Molecule Real Time
   **PCR**: Polymerase Chain Reaction
   **DNA**: Deoxyribonucleic acid
   **DNase**: Deoxyribonuclease
   **SOP**: Standard Operating Procedure
   **BHI**: Brain Heart Infusion
   **RVS**: Rappaport-Vassiliadis soya
   **XLD**: Xylose lysine deoxycholate
   **BGA**: Brilliant green agar
   **TT**: Tetrathionate
   **BSA**: Bismuth sulphite agar
   **bp**: base pairs
   **PGAP**: Prokaryotic Genome Annotation Pipeline
   **RAST**: Rapid Annotation using Subsystem Technology
4. **Health and Safety**: Foodborne pathogens and human and environmental biological samples can cause serious disease. Always a minimum of Biosafety level 2 practices and use extreme caution when transferring strains and biological samples of this type.

## Supplementary Figures

**Supplementary Figure 1: Principal component analysis (PCA) of microbial community composition across sample sources (n=139).** Principal component analysis (PCA) plots showing the ordination of samples based on genus-level relative abundances for each of the nine sample sources. Samples are stratified according to sequencing library preparation method: blue points denote samples prepared using PCR-free KAPA Hyper Prep kit, and green points denote minimal PCR kit. Arrows indicate the top 15 taxa contributing most strongly to the ordination space, with arrow direction representing the gradient of increasing taxon abundance and arrow length corresponding to the strength of contribution to the principal components.

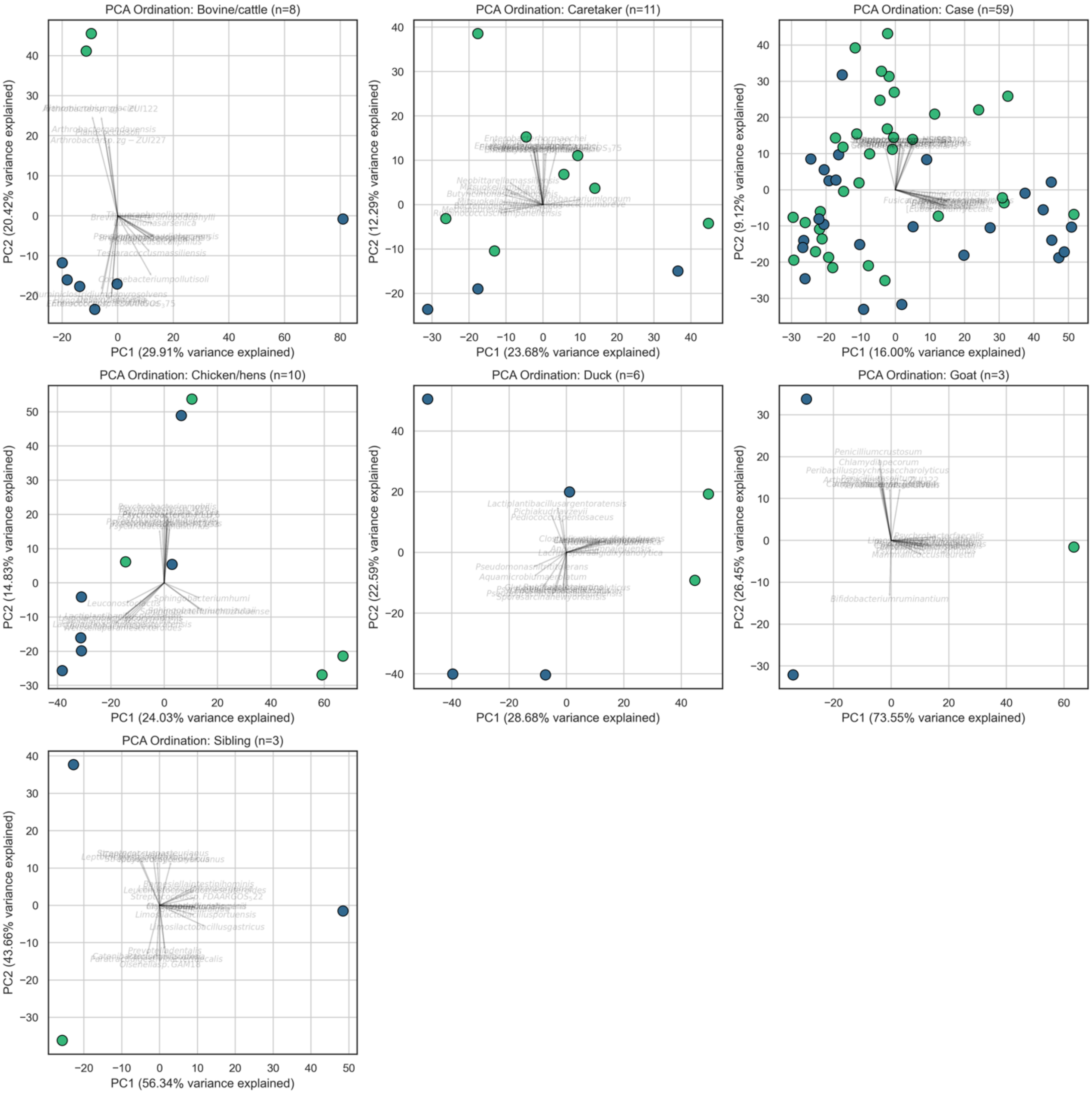

**Supplementary Tables**

**Supplementary Table 1: PERMANOVA pseudo-F table showing the results of the PCR free and PCR amplification process.** PERMANOVA pseudo-F table showing the results of the analysis of the relationship between PCR free and PCR amplification for samples which had been sequenced using both. Rows in bold shows the isolation source which were statistical significant. SS = Sum of squares, MS = Mean Squares.

| | **Number of samples** | **df** | **SS** | **Pseudo-F** | **p-val** | **FDR-adjusted p-val** |
|---|---|---|---|---|---|---|
| Caretaker | 11 | 1 | 6,018 | 1.0702 | 0.258 | 0.3582 |
| Case | 59 | 1 | 30,831 | 1.9426 | 0.007 | 0.049 |
| Goat | 3 | 1 | 24,229 | 2.7684 | 0.307 | 0.3582 |
| Bovine/cattle | 8 | 1 | 13,538 | 1,5548 | 0.073 | 0.1278 |
| Chicken/hens | 10 | 1 | 19,442 | 1.5172 | 0.029 | 0.1015 |
| Duck | 6 | 1 | 17,268 | 1.4433 | 0.069 | 0.1278 |
| Sibling | 3 | 1 | 5,623 | 0.8949 | 0.672 | 0.672 |
