# Supplementary material for: Using metagenomics and whole-genome sequencing to characterize enteric pathogens across various sources in Africa
Source: Nat Commun. 2025 Nov 28;16:11311. doi: 10.1038/s41467-025-66400-9 (PMC12722760; doi:10.1038/s41467-025-66400-9)
Supplement: Supplementary file 2 — Description of Additional Supplementary Files [file 41467_2025_66400_MOESM2_ESM.pdf]

Description of Additional Supplementary Files

Supplementary Data 1.
Samples collection information and metadata for whole genome sequencing (WGS) isolates and metagenomic reads.
